# Pregnancy Induces Transcriptional Activation of the Peripheral Innate Immune System and Increases Oxidative DNA Damage among Healthy Third Trimester Pregnant Women

**DOI:** 10.1371/journal.pone.0046736

**Published:** 2012-11-02

**Authors:** Xinyin Jiang, Haim Y. Bar, Jian Yan, Allyson A. West, Cydne A. Perry, Olga V. Malysheva, Srisatish Devapatla, Eva Pressman, Francoise M. Vermeylen, Martin T. Wells, Marie A. Caudill

**Affiliations:** 1 Division of Nutritional Sciences, Cornell University, Ithaca, New York, United States of America; 2 Cornell University Statistical Consulting Unit, Cornell University, Ithaca, New York, United States of America; 3 Department of Pediatrics, Cayuga Medical Center, Ithaca, New York, United States of America; 4 Obstetrics and Gynecology, University of Rochester Medical Center, Rochester, New York, United States of America; 5 Department of Statistical Science, Cornell University, Ithaca, New York, United States of America; Emory University School of Medicine, United States of America

## Abstract

**Background:**

Pregnancy induces physiological adaptations that may involve, or contribute to, alterations in the genomic landscape. Pregnancy also increases the nutritional demand for choline, an essential nutrient that can modulate epigenomic and transcriptomic readouts secondary to its role as a methyl donor. Nevertheless, the interplay between human pregnancy, choline and the human genome is largely unexplored.

**Methodology/Principal Findings:**

As part of a controlled feeding study, we assessed the influence of pregnancy and choline intake on maternal genomic markers. Healthy third trimester pregnant (n = 26, wk 26–29 gestation) and nonpregnant (n = 21) women were randomized to choline intakes of 480 mg/day, approximating the Adequate Intake level, or 930 mg/day for 12-weeks. Blood leukocytes were acquired at study week 0 and study week 12 for microarray, DNA damage and global DNA/histone methylation measurements. A main effect of pregnancy that was independent of choline intake was detected on several of the maternal leukocyte genomic markers. Compared to nonpregnant women, third trimester pregnant women exhibited higher (*P<0.05*) transcript abundance of defense response genes associated with the innate immune system including pattern recognition molecules, neutrophil granule proteins and oxidases, complement proteins, cytokines and chemokines. Pregnant women also exhibited higher (*P*<0.001) levels of DNA damage in blood leukocytes, a genomic marker of oxidative stress. No effect of choline intake was detected on the maternal leukocyte genomic markers with the exception of histone 3 lysine 4 di-methylation which was lower among pregnant women in the 930 versus 480 mg/d choline intake group.

**Conclusions:**

Pregnancy induces transcriptional activation of the peripheral innate immune system and increases oxidative DNA damage among healthy third trimester pregnant women.

## Introduction

Pregnancy induces several physiological adaptations to meet the needs of the developing fetus and the health requirements of the mother. Many of these pregnancy-induced physiologic adaptations may involve, or contribute to, alterations in the genomic landscape. Nevertheless, very few studies have examined the interplay between pregnancy and the human genome.

A prominent change during pregnancy is the modulation of the immune system to accommodate the presence of a semiallogeneic fetus [1]. In normal pregnancy, there is a shift in the balance of T lymphocytes, mediators of the adaptive cellular immune response, towards a Th2 (immunosuppressive) phenotype with an anti-inflammatory cytokine profile. However, there is also a need to defend against pathogens for the mother-fetus dyad [2]. Neutrophils, mediators of the innate immune response that play a key role in the first line of defense against pathogens, increase in peripheral blood during pregnancy [3]. Nevertheless, it is unclear as to whether these neutrophils are activated. *In vitro* studies showed that neutrophils from pregnant women exhibited reduced microbial killing and chemotaxis [4], [5]. However, flow cytometry studies showed that several neutrophil surface markers were higher in the pregnant state indicating neutrophil activation [6], [7]. Thus, studies that profile the transcriptome of blood leukocytes are needed to better understand the status of the immune system during human pregnancy.

A greater susceptibility to oxidative stress, mostly because of the mitochondria-rich placenta [8], is another characteristic of human pregnancy. Oxidative stress can cause DNA damage [9], which in turn can lead to aberrant gene expression and apoptosis. Although higher levels of DNA damage are detected among women with complicated pregnancies [9], [10], it is unclear whether DNA damage is elevated in normal pregnancy. Studies that examine indicators of DNA damage are needed to advance understanding of redox balance during normal pregnancy and to inform nutritional therapeutic opportunities.

Pregnancy is also associated with an increased demand for methyl donors to maintain epigenetic marks in the expanding maternal and fetal tissues [11]. DNA and histone methylation play a fundamental role in regulating chromatin structure, stability, and gene expression [12], [13]. A major source of methyl groups for DNA and histone methylation is the essential micronutrient choline, which is recommended at an intake level of 450 mg/d during pregnancy [14]. Notably, we previously demonstrated in third trimester pregnant women that consumption of choline at approximately two times the current intake recommendation (i.e., 930 versus 480 mg/d) substantially altered epigenetic marks [15] and transcriptome readouts (unpublished data) in fetal derived tissues (i.e., placental and cord blood leukocytes). Nonetheless, it remains to be determined if a maternal choline intake exceeding current recommendations can alter genomic marks in the mothers themselves.

The aims of the current study were to investigate the influence of both pregnancy and maternal choline intake on leukocyte genomic markers. To accomplish these aims, we examined genome-wide gene expression, DNA damage, and global DNA/histone methylation in blood leukocytes obtained from third trimester pregnant women and nonpregnant control women enrolled in a 12-week controlled feeding study [16].

## Materials and Methods

### Study Participants

Healthy third trimester (week 26–29 gestation) singleton pregnant women and nonpregnant control women aged ≥21 y were recruited from Ithaca, New York, and surrounding areas between January 2009 and October 2010. Entry into the study was contingent upon good health status, no tobacco or alcohol use, and a willingness to comply with the study protocol. Twenty-six of the 29 pregnant women and 21 of the 22 nonpregnant women who started the study completed it. Information regarding the study participants and the CONSORT flowchart have been reported previously [16].

### Description of procedures

#### Study Design and Diet

This was a 12-week controlled feeding study in which pregnant (n = 26) and nonpregnant (n = 21) women were randomized to either 480 (an intake level that approximates the choline adequate intake, AI) or 930 mg choline/d. The choline was derived from the diet (380 mg/d) plus supplemental choline chloride (either 100 or 550 mg choline/d for the 480 or 930 mg/d intake levels, respectively). Throughout the 12-wk study, the participants consumed a 7-day cycle menu. Choline-free prenatal multivitamins and docosahexaenoic acid supplements were provided daily as detailed in Yan et al [16]. The fact that all participants consumed the same diet and supplements makes this study particularly suitable to examining the effects of pregnancy on a wide-array of genomic and metabolic endpoints.

#### Sample collection

Blood samples were obtained at study-baseline (week 0) and study-end (week 12). Fasting (10-h) peripheral blood mononuclear cells (PBMC, including lymphocytes and monocytes) samples were retrieved with Vacutainer CPT tubes (BD, Franklin Lakes, NJ); whole blood, leukocyte and plasma samples were retrieved with EDTA tubes (BD) as previously described [16], [17].

#### Analytical measurements

*Complete blood counts* were conducted with 1 mL whole blood using an AcT diff 2 hematology analyzer (Beckman Coulter, Brea, CA) according to the manufacturer's instructions.

*Total RNA purification* was performed with a commercially available kit (RNeasy Mini kit, Qiagen, Valencia, CA) as previously detailed [15]. The integrity of the RNA samples was determined using an Agilent 2100 Bioanalyzer (Agilent Technologies, Santa Clara, CA). Only samples of an RNA Integrity Number (RIN) >8.0 were included for microarray. There were 12 sets (a set includes both a wk-0 and a wk-12 measurement) of samples from pregnant participants (n =  6/choline intake group) and 10 sets of samples from nonpregnant participants (n = 5/choline intake group) that passed the RIN cutoff.

*Gene expression profiling* was performed using the Whole Human Gene Expression Microarray 4×44 K (Agilent). Antisense RNA amplification and cyanine-3 labeling was performed with an Amino Allyl MessageAmp II aRNA amplification kit (Ambion Inc., Grand island, NY). Microarray hybridization was performed at 65°C for 17-h in an Agilent Microarray Hybridization oven. Microarray scanning was performed in an Agilent Scanner (G2505C) and the images were extracted using Agilent Feature Extraction Software 10.5. Data normalization was performed using log2 transformation and median normalization. An extension of the Laplace approximation EM Microarray Analysis (LEMMA) package based on the R software platform (http://www.R-project.org) [18] developed by Bar et al. [19], [20] was used to detect differentially expressed genes. The version used here (unpublished data) can sensitively detect differentially expressed genes, as it takes into account not only mean differences, but also variational differences between the comparison groups. Using this method we were able to increase the number of discoveries of differentially expressed genes, relative to other methods that do not account for differential variation. The differentially expressed genes were declared by controlling the false discovery rate (FDR) with Benjamini-Hochberg (BH) correction <0.05 and expression fold difference >2. Differentially expressed genes were classified according to their gene ontology (GO) using High-Throughput GoMiner [21]. Hierarchical clustering was conducted with MultiExperiment Viewer [22], [23]. Microarray results of select genes were verified using quantitative real-time PCR. All data is MIAME compliant. The data has been deposited in a MIAME compliant database NCBI's Gene Expression Omnibus [24] as detailed on the MGED Society website (http://www.mged.org/Workgroups/MIAME/miame.html) and are accessible through GEO Series accession number GSE36532 (http://www.ncbi.nlm.nih.gov/geo/query/acc.cgi?acc=GSE36532).

*Reverse transcription* and *quantitative real-time PCR* were performed as previously described [25]. Data are expressed using the delta delta Ct method [26] and glyceraldehyde-3-phosphate dehydrogenase (*GAPDH*) is used as the housekeeping gene.

*Circulating tumor necrosis factor alpha (TNFα) and interleukin 6 (IL6)* were measured in EDTA plasma with commercially available ELISA kits (R&D systems, Minneapolis, MN) according to the manufacturer's instructions.

*Peripheral blood mononuclear cell (PBMC) DNA damage* was measured in duplicate via the alkaline version of single-cell gel electrophoresis (COMET assay). Sample preparation was performed as described previously [17]. DNA migration from the nucleus was visualized (75 cells/sample) with an Olympus BX-50 light microscope and a high resolution QImaging Retiga EXi cooled CCD camera. Photos were acquired using the MetaMorph Premier (ver. 7.0) software (Molecular Devices, Sunnyvale, CA) after staining with SYBR Gold fluorescent dye (Invitrogen, Grand Island, NY). Percent tail DNA (defined as the proportion of DNA that has migrated from the nucleus) was used as an index of DNA damage and was calculated using the software Komet 6.0 (Andor Technology, South Windsor, CT).

*Global DNA methylation* was measured in leukocytes using liquid chromatography-tandem mass spectrometry (LC-MS/MS) as described by Song et al. [27] with modifications based on our instrumentation [17]. Global DNA methylation [5-methyl-2′-deoxycytidine (5mdC)] is expressed as a percentage of 2′-deoxyguanosine (representing total 2′-deoxycytidine).

*Global histone methylation* was measured with western blots as described previously [15]. Leukocyte samples (200 µL) were suspended in 0.2 N hydrochloric acid at 4°C overnight. The extracts were separated by SDS-PAGE and transferred to Immobilon-FL polyvinylidene fluoride transfer membranes. Membranes were incubated with corresponding primary and secondary antibodies [15]. Target protein bands were quantified with the LI-COR Odyssey® imaging system (LI-COR, Lincoln, NE) and expressed as the ratio of intensity of the histone epigenetic mark to the reference total histone 3 proteins.

### Ethics

The study was approved by the Cornell University and Cayuga Medical Center Institutional Review Boards for Human Participants and written informed consent was obtained from every participant prior to study entry. The study was registered at clinicaltrials.gov as NCT01127022.

### Statistical analysis

The effect of pregnancy on the dependent variables (e.g. DNA damage, epigenetic marks, gene expression) was assessed at study-baseline (wk-0) and study-end (wk-12) separately with general linear models (GLMs). Pregnancy status and choline intake were included as independent variables and all two-way interactions were tested. The effect of choline intake on the genomic markers was assessed at study-end in a similar manner with study-baseline data included as a covariate. All of the analyses included leukocyte sub-population (e.g. the percentage of granulocytes) as a covariate with the exception of the DNA damage variable which only contains PBMCs. Additional candidates for entry as covariates into the statistical models are listed in Table 1. Covariates and interaction terms that did not achieve a statistical significance of *P*≤0.05 were removed from the models in a stepwise process.

**Table 1:** Baseline (study week 0) characteristics of third trimester pregnant women and nonpregnant women randomized to either the 480 or 930 mg/d choline intake group.

	Pregnant		Nonpregnant	
	480 mg/d (n = 13)	930 mg/d (n = 13)	480 mg/d (n = 10)	930 mg/d (n = 11)
Age, y (range)	29 (25–33)	28 (22–34)	28 (21–37)	29 (21–40)
Ethnicity, Caucasian/African American/Latino/Asian/Other	9/0/2/1/1	7/1/2/3/0	8/1/1/0/0	6/1/1/1/2
BMI, kg/m^2^ (range)^a^	23.6 (20.2–31.9)	23.6 (19.9–29.8)	23.6 (19.6–27.3)	23.5 (18.2–29.8)
Education, high school/college	1/12	3/10	3/7	3/8
Physical activity, usual daily activity/exercise ≥3 times per wk/unknown	5/7/1	1/10/2	2/8/0	1/9/1
Vitamin supplement consumption, yes/no^b^	11/2	11/2	2/8	5/6

a For pregnant women, this parameter represents pre-pregnancy BMI.

b *P*<0.01 between pregnant and nonpregnant women, Chi-square test.

Plots and histograms of the residuals were used to assess normality in the models. Dependent variables that deviated from the normal distribution (e.g. global histone methylation and quantitative PCR data) were logarithmically transformed to meet the assumption of normality. Differences were considered to be significant at *P*≤0.05; *P*<0.10 was considered to be indicative of trends. Values are presented as means ± SEM. If covariates were retained in the final models, the values presented are predicted means; *P* values are two-tailed. All analyses were performed using SPSS (release 18.0 for Windows, SPSS Inc, Chicago, IL).

## Results

### Participant characteristics

The characteristics of the study population at study-baseline (wk-0) are shown in Table 1. Third trimester pregnant and nonpregnant women did not differ in their age, ethnicity/race, pre-pregnancy BMI, education, or physical activity. However, vitamin supplement consumption was greater (*P*<0.01) among pregnant women. No differences (*P*>0.13) in the baseline characteristics were detected among the choline intake groups (Table 1). All pregnant women delivered their babies at term without major complications and their babies were apparently healthy [16].

### The effects of choline

**Figure 1:**
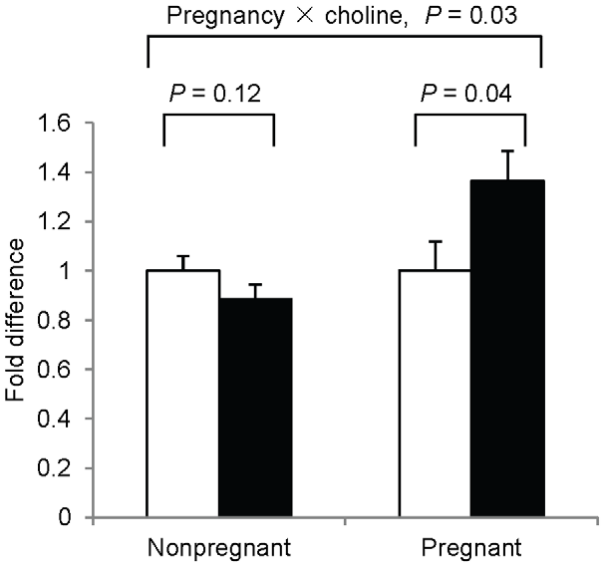
Effect of choline intake on peripheral blood leukocyte H3K4me2. The relative abundance of H3K4me2 at study-end in third trimester pregnant women (right) and nonpregnant women (left) consuming 930 versus 480 mg choline/d. White bar: 480 mg choline/d group, black bar: 930 mg choline/d group. n = 10–13/choline intake and pregnancy status. Values are predicted means ± SEM. Analyzed with general linear models.

No main effects of choline (P>0.05) were detected for any of the dependent variables. However, choline intake interacted (*P* = 0.03) with pregnancy status to affect H3K4me2. After stratifying by pregnancy status, H3K4me2 was higher (*P* = 0.04, controlling for the leukocyte sub-populations) in the 930 versus 480 mg choline/d group among pregnant women (Figure 1). This relationship did not achieve statistical significance among nonpregnant women (*P* = 0.12). No additional choline × pregnancy interactions (*P*>0.05) were detected.

### The effects of pregnancy

A main effect of pregnancy was detected for several of the dependent variables at study-baseline and study-end (values taken after controlled feeding and choline randomization) as described in subsequent text. The pregnancy effect was not modified by choline intake (i.e., no significant pregnancy × choline interactions were detected for any of the variables) at study-end indicating an effect of pregnancy that is independent of choline intake.

#### Peripheral blood leukocyte counts

Granulocytes and monocytes are important components of the innate immune system. Third trimester pregnant women exhibited higher (*P*<0.01) granulocyte counts (the majority of which was neutrophils) and borderline higher (*P*<0.09) monocyte counts than nonpregnant women at both study-baseline and study-end. Lymphocyte count was not altered by pregnancy (Table 2).

**Table 2:** Peripheral blood leukocyte counts in third trimester pregnant women and nonpregnant women at the beginning and end of the controlled feeding study.

	Study-baseline			Study-end		
	Pregnant	Nonpregnant	*P* value	Pregnant	Nonpregnant	*P* value
	(n = 26)	(n = 21)		(n = 26)	(n = 21)	
Leukocytes (×10^3^/µL)	9.8±0.5	6.1±0.2	< 0.01	9.7±0.5	6.3±0.3	< 0.01
Lymphocytes (×10^3^/µL)	1.8±0.1	1.9±0.1	0.30	2.0±0.1	2.1±0.1	0.70
Monocytes (×10^3^/µL)	0.3±0.03	0.2±0.02	< 0.01	0.3±0.02	0.2±0.02	0.09
Granulocytes (×10^3^/µL)	7.7±0.4	4.0±0.2	< 0.01	7.4±0.4	3.9±0.2	< 0.01
Granulocyte (%)	78.2±0.9	65.4±1.4	< 0.01	75.4±0.9	62.5±1.6	< 0.01

Data were analyzed using general linear models.

#### Leukocyte genome wide expression

**Figure 2:**
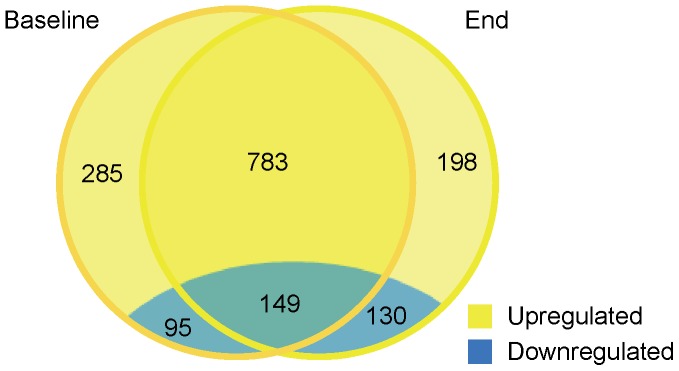
Venn diagram of differentially expressed genes by pregnancy. This figure presents the number of genes differentially expressed in third trimester pregnant versus nonpregnant women at study-baseline (circle on the left) and study-end (circle on the right). The number of genes altered both at study-baseline and study-end are presented in the intersecting area of the two circles. Color scheme: blue represents low expression and yellow represents high expression. n = 12 for pregnant women; n = 10 for nonpregnant women. Analyzed with the LEMMA statistical package.

There were 1068 upregulated and 244 downregulated genes at study-baseline, and 1048 upregulated and 280 downregulated genes at study-end, in pregnant versus nonpregnant women (Figure 2). Of these ∼1300 differentially expressed genes, 932 were altered at both study-baseline and study-end (Table S1). The differential expression of select genes was verified by quantitative PCR (Table 3). Gene expression did not differ between study-end and study-baseline, suggesting that the leukocyte gene expression profile was stable throughout the third trimester of pregnancy.

**Table 3:** Quantitative PCR verification of microarray results of genes differentially expressed in third trimester pregnant women (n = 12) and nonpregnant women (n = 10) at the end of the controlled feeding study.

Gene symbol	Gene name	Function	Total transcript abundance		Transcript abundance/percent granulocyte		Transcript abundance/granulocyte count	
			Fold change	*P* value	Fold change	*P* value	Fold change	*P* value
*OLFM4*	Olfactomedin 4	Cell adhesion	64	<0.01	49	<0.01	32	<0.01
*LTF*	Lactoferrin	Host defense	39	<0.01	30	<0.01	23	<0.01
*ELANE*	Elastase	Virulence factor degradation	34	<0.01	26	<0.01	18	<0.01
*DEFA4*	Defensin, alpha 4	Host defense	25	<0.01	20	<0.01	15	<0.01

Data were analyzed with general linear models. Fold change was calculated as the average transcript abundance in pregnant women/the average transcript abundance in nonpregnant women.

**Figure 3:**
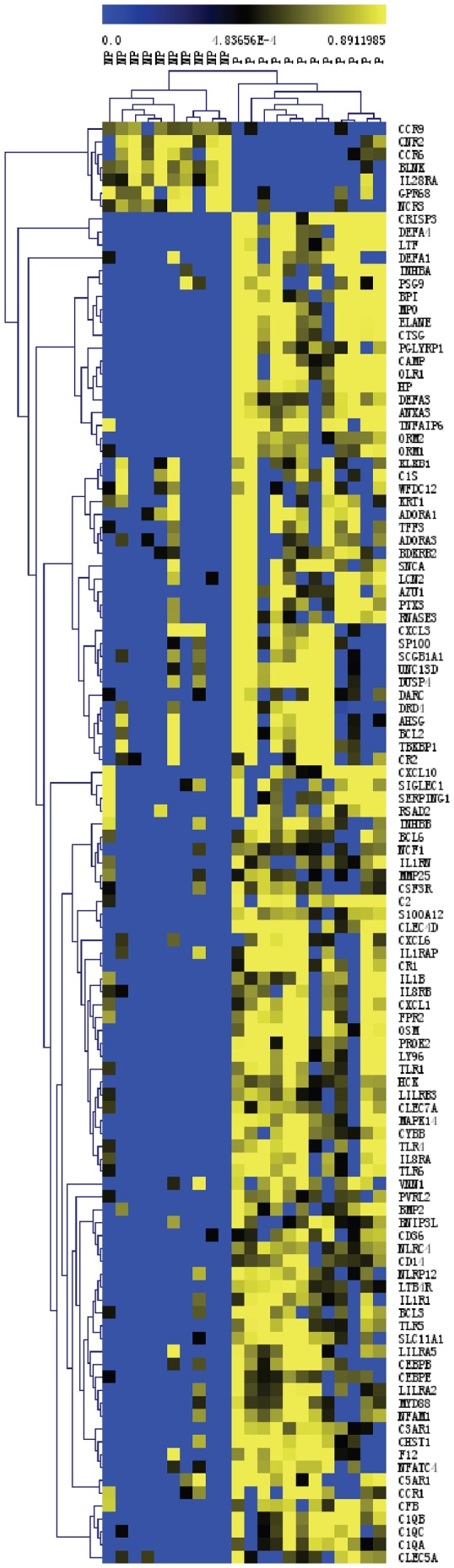
Hierarchical clustering of differentially expressed immune defense genes (GO: 0006952) in pregnant versus nonpregnant women. This figure presents the hierarchical clustering of 112 differentially expressed immune defense genes in third trimester women versus nonpregnant women (reference group) at the beginning and end of the controlled feeding study. Color scheme: blue represents low expression and yellow represents high expression. n = 12 for pregnant women; n = 10 for nonpregnant women. Analyzed with Euclidean distances using MultiExperiment Viewer.

The GO analyses conducted at study-baseline and study-end showed similar biological themes: thus, only the GO results at study-end are presented. At study-end, 246 molecular function categories were altered (*P*<0.01) (Table S2), ∼40% of which was related to immune response. The GO of defense response (GO: 0006952) showed the most significant alteration. Of the 112 defense response genes altered, 105 were upregulated in third trimester pregnant women as compared to nonpregnant women (Figure 3). To evaluate whether the elevated expression of defense response genes was mediated at least in part by the activation of granulocytes, we used leukocyte sub-population as a covariate in the statistical models. After adjusting for leukocyte sub-populations, the greater expression of the defense response genes among pregnant women was maintained (examples are shown in Table 3 and 4), suggesting that the higher expression of defense response genes was not solely a function of higher granulocyte counts, but also due to enhanced activation of the granulocytes.

**Table 4:** Select list of genes involved in defense response that were upregulated in third trimester pregnant women at the end of the controlled feeding study.

Gene symbol	Gene name	Category	Fold change (unadjusted)	Fold change (adjusted)
*CD14*	CD14 molecule	Surface marker	2.30	1.77
*TLR1*	Toll-like receptor 1	TLR signaling pathway	2.16	1.79
*TLR4*	Toll-like receptor 4	TLR signaling pathway	2.47	1.94
*TLR5*	Toll-like receptor 5	TLR signaling pathway	3.07	2.43
*TLR6*	Toll-like receptor 6	TLR signaling pathway	2.51	1.97
*MYD88*	Myeloid differentiation primary response gene (88)	TLR signaling pathway	2.02	1.51
*ELANE*	Elastase, neutrophil expressed	Azurophil granule protein	11.05	13.77
*MPO*	Myeloperoxidase	Azurophil granule protein	7.25	7.44
*AZU1*	Azurocidin 1	Azurophil granule protein	2.94	2.93
*CTSG*	Cathepsin G	Azurophil granule protein	7.50	7.03
*DEFA1*	Defensin, alpha 1	Azurophil granule protein	4.76	3.72
*DEFA3*	Defensin, alpha 3, neutrophil-specific	Azurophil granule protein	7.53	4.97
*DEFA4*	Defensin, alpha 4, corticostatin	Azurophil granule protein	26.95	25.81
*LTF*	Lactotransferrin	Specific granule protein	35.51	26.33
*CAMP*	Cathelicidin antimicrobial peptide	Specific granule protein	17.48	15.59
*C1QB*	Complement component 1, q subcomponent, B	Complement system	3.35	2.62
*CR1*	Complement component (3 b/4 b) receptor 1	Complement system	3.20	2.50
*C2*	Complement component 2	Complement system	3.58	2.80
*IL1B*	Interleukin 1, beta	Cytokine signaling	2.78	2.14
*IL1R1*	Interleukin 1 receptor, type I	Cytokine signaling	2.42	1.77
*IL8RB*	Interleukin 8 receptor, beta	Cytokine signaling	2.86	2.23
*IL8RA*	Interleukin 8 receptor, alpha	Cytokine signaling	2.81	2.19

*P* values<0.05 with or without adjusting for leukocyte sub-populations as analyzed with general linear models or the LEMMA statistical package, respectively. Fold change was calculated as the average transcript abundance in pregnant women/the average transcript abundance in nonpregnant women.

Among pregnant women, several components of the toll-like receptor (TLR) signaling pathway were upregulated, including the genes encoding TLR1, 4, 5, 6; the CD molecule 14 (*CD14*), a major surface marker of neutrophils and monocytes that interacts with TLR4; and myeloid differentiation primary response gene 88 (*MYD88*). The TLRs are pattern recognition molecules on immune cells (e.g., neutrophils) that recognize pathogenic elements (e.g., lipopolysaccharides) and illicit a signaling cascade involving MYD88 that results in the increased production of proinflammatory cytokines such as interleukin 1β (IL1β) and the chemokine interleukin 8 (IL8) [28], [29]. The increased IL8 production favors chemotaxis and survival of the neutrophils [30].

Third trimester pregnant women also exhibited higher expression of genes encoding: (i) the microbicidal proteins stored in the neutrophil granules [e.g. the azurophil proteins elastase (*ELANE*), alpha-defensins (*DEFA*) and specific granule proteins lactoferrin (*LTF*) and catheticidin (*CAMP*)]; (ii) the complement proteins involved in innate humoral immune response [e.g. complement component 1, q subcomponent, B chain (*C1QB*), complement component 2 (*C2*) and complement component (3 b/4 b) receptor 1 (*CR1*)]; (iii) proteins involved in neutrophil superoxide anion production [e.g. the NADPH oxidase complex neutrophil cytosolic factors (*NCF*), and myeloperoxidase (*MPO*)]; and (iv) the proinflammatory cytokines (e.g. *IL1B*). The GO of TNF (GO:0032640) and IL6 (GO:0032675) production were also upregulated.

Only a few (∼8) immune response genes were downregulated among pregnant women after controlling for the leukocyte sub-populations. The repressed genes were mostly associated with the adaptive immune response (Table 5) and included: (i) the transmembrane glycoprotein CD1 family members CD1c and CD1e molecules, which present primary lipid and glycolipid antigens to T cells [31]; (ii) the chemokine (C-C motif) receptors *CCR6* and *CCR9*, which are preferably expressed by T cells [32], [33]; (iii) CD70 molecule, a surface antigen on activated T and B lymphocytes [34] and (iv) B-cell linker protein (*BLNK*), which plays an important role in B cell development and receptor signaling [35].

**Table 5:** Genes involved in adaptive immune response that were downregulated in third trimester pregnant women at the end of the controlled feeding study.

Gene symbol	Gene name	Function	Fold change (unadjusted)	Fold change (adjusted)
*CD70*	CD70 molecule	Activated T and B cell marker	0.47	0.61
*CD1C*	CD1c molecule	Antigen presentation	0.46	0.63
*CD1E*	CD1e molecule	Antigen presentation	0.42	0.55
*CCR6*	chemokine (C-C motif) receptor 6	T cell recruitment	0.34	0.54
*CCR9*	chemokine (C-C motif) receptor 9	T cell recruitment	0.50	0.47
*BLNK*	B cell linker	B cell receptor signaling	0.38	0.47

*P* values<0.05 with or without adjusting for leukocyte sub-populations as analyzed with general linear models or the LEMMA statistical package, respectively. Fold change was calculated as the average transcript abundance in pregnant women/the average transcript abundance in nonpregnant women.

#### Plasma TNFαand IL6

**Figure 4:**
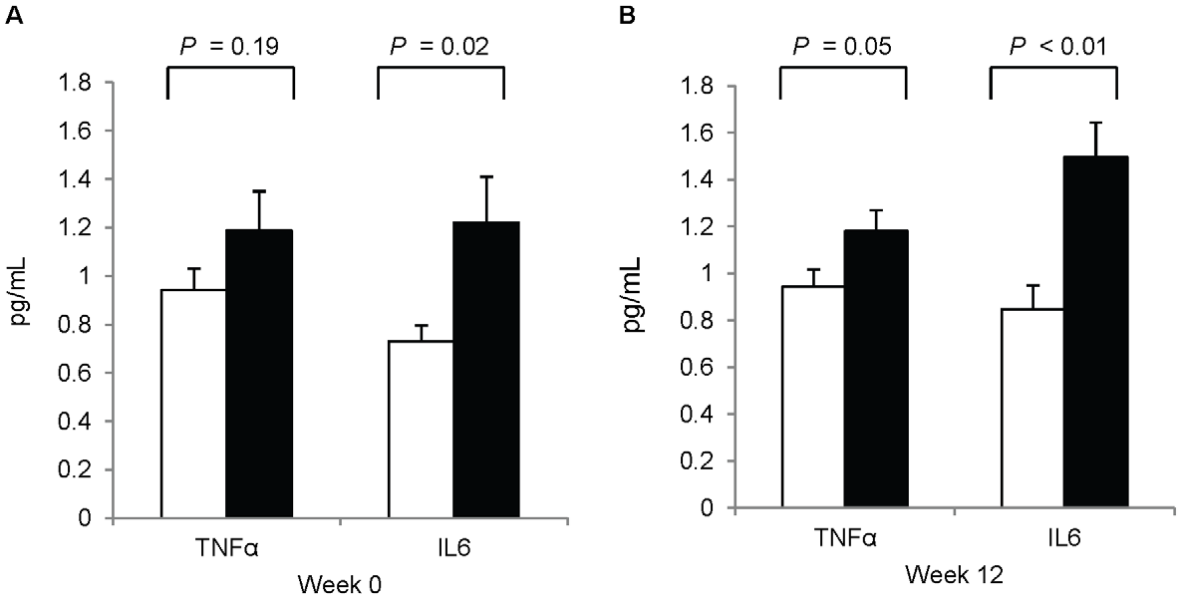
Plasma TNFα and IL6 concentrations. Plasma concentrations of TNFα and IL6 in third trimester pregnant versus nonpregnant women at study-baseline (**A**) and study-end (**B**). White bar: nonpregnant women (n = 21); black bar: pregnant women (n = 26). Values are means ± SEM. Analyzed with general linear models.

Circulating concentrations of TNFα were higher among pregnant women at study-end (1.18±0.09 vs. 0.94±0.07 pg/mL, *P* = 0.05) but not at study-baseline (1.19±0.16 vs. 0.94±0.09 pg/mL, *P* = 0.19). IL6 was elevated among pregnant women at both study-baseline (1.22±0.19 vs. 0.73±0.07 pg/mL, *P* = 0.02) and study-end (1.50±0.15 vs. 0.73±0.10 pg/mL, *P*<0.01) (Figure 4). The higher circulating concentrations of TNFα and IL6 among pregnant women at study-end corresponded with the microarray GO results showing upregulation of TNF and IL6 production.

#### Leukocyte percent tail DNA

Third trimester pregnant women had significantly higher (*P*<0.01) percent tail DNA than nonpregnant women at study-baseline and study-end (Table 6). The higher percent tail DNA represents greater DNA strand breakage due to oxidative stress.

**Table 6:** Leukocyte DNA damage and global DNA methylation in third trimester pregnant women and nonpregnant women consuming 480 or 930 mg choline/d at the beginning and end of the controlled feeding study.

	Pregnant			Nonpregnant		
	480 mg/d	930 mg/d	Total	480 mg/d	930 mg/d	Total
	(n = 13)	(n = 13)	(n = 26)	(n = 10)	(n = 11)	(n = 21)
***Percent tail DNA***						
Study-beginning	44.9±3.6	43.5±4.8	44.3±2.9*	26.4±2.9	27.8±4.0	27.1±2.5
Study-end	33.2±4.6	43.5±4.8	38.3±3.4*	20.6±1.9	24.9±1.5	22.9±1.6
***DNA methylation***						
Study-beginning	5.1±0.2	5.2±0.2	5.1±0.1	4.9±0.2	5.2±0.2	5.1±0.1
Study-end	5.2±0.2	5.1±0.2	5.1±0.1	5.0±0.1	4.8±0.2	4.9±0.1

Data were analyzed with general linear models.

* *P*<0.01 between pregnant and nonpregnant women.

#### Leukocyte global histone and DNA methylation

**Figure 5:**
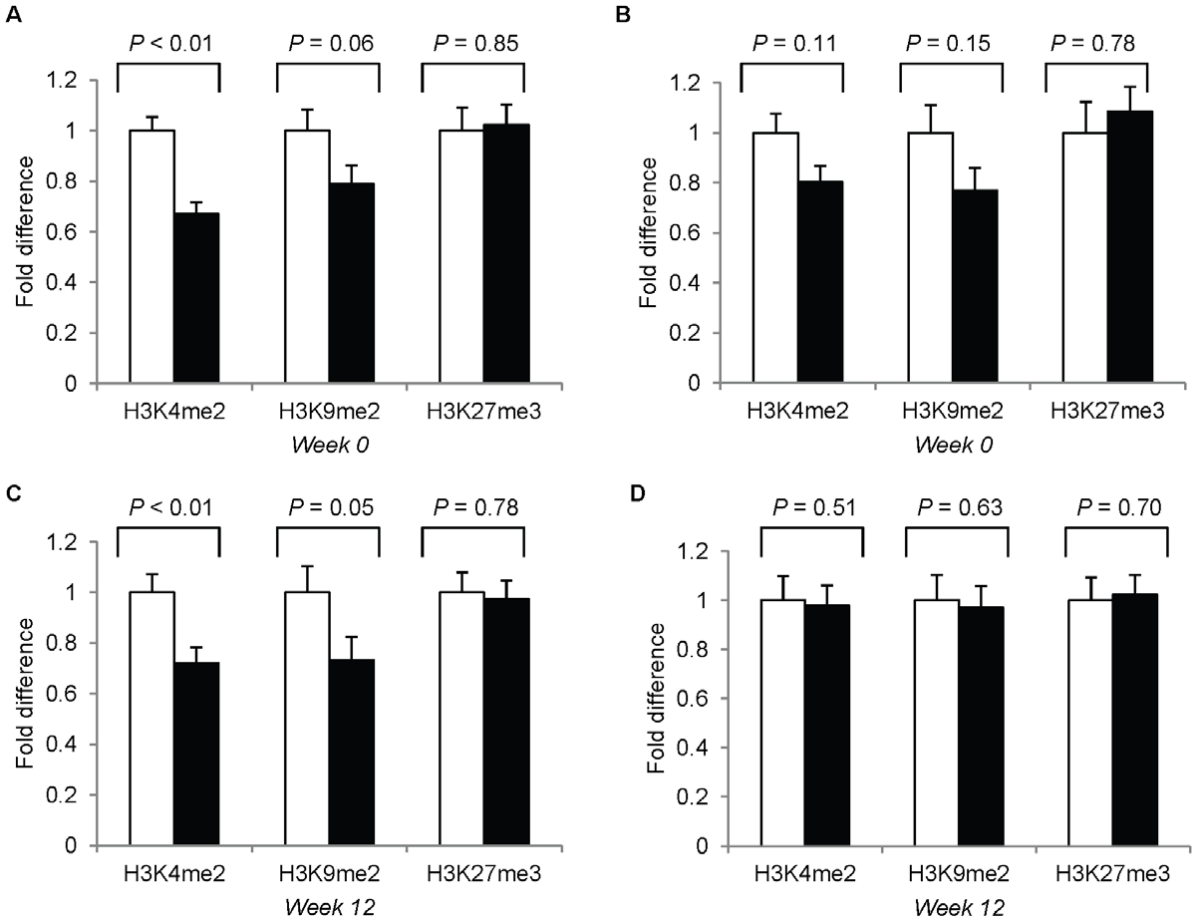
Peripheral blood leukocyte histone modification marks H3K4me2, H3K9me2 and H3K27me3. (**A**) and (**C**), histone modifications in third trimester pregnant versus nonpregnant women without controlling for percent granulocytes at study-baseline and study-end, respectively; (**B**) and (**D**), histone modifications in third trimester pregnant versus nonpregnant women controlling for percent granulocytes at study-baseline or study-end, respectively. White bar: nonpregnant women (n = 21), black bar: pregnant women (n = 26). Data are predicted means ± SEM. Analyzed with general linear models.

Third trimester pregnant women (versus nonpregnant women) had lower levels of the transcription activation histone mark H3K4me2 (*P*<0.01), and the transcription repression mark H3K9me2 (*P* = 0.05) at study-baseline and study-end (not controlling for leukocyte sub-populations) (Figure 5, A and C). However, after controlling for leukocyte sub-populations, the effect of pregnancy disappeared (Figure 5, B and D), suggesting that the alterations in leukocyte H3K9me2 and H3K4me2 during pregnancy were a function of the shift in the leukocyte sub-populations and that different leukocyte cell types may have a different abundance of these histone modifications. Global DNA methylation (study-baseline or study-end) was not altered (P>0.38) by pregnancy (Table 6).

## Discussion

Genomic markers were investigated among healthy third trimester pregnant and nonpregnant control women to advance our understanding of pregnancy induced alterations in immune response, redox balance, and epigenomic stability. The effect of maternal choline intake on these genomic markers was also investigated secondary to the role of choline as a methyl donor. Under both uncontrolled (study-baseline) and controlled (study-end) dietary conditions, our data demonstrate that healthy third trimester pregnant women experience transcriptional activation of the peripheral innate immune system and elevated oxidative stress as indicated by higher DNA damage. Our data also indicate that a choline intake exceeding current dietary recommendations does not alter blood leukocyte genomic markers, except for H3K4me2 among third-trimester pregnant women.

### The effects of pregnancy

#### Immune Function

Pregnant women experienced an elevation in the transcript abundance of 105 leukocyte defense response genes as compared to nonpregnant control women. The majority of upregulated defense genes were neutrophil-associated genes including neutrophil surface markers (e.g. *CD14*), pattern recognition molecules, cytokines, chemokines, neutrophil granule proteins, complement proteins and neutrophil oxidases. Importantly, the greater transcription of these host defense genes among third trimester pregnant women persisted after controlling for the leukocyte sub-populations, indicating transcriptional activation, rather than suppression, of individual neutrophils. The activation of peripheral neutrophils (and the innate immune system) during the last third of human pregnancy may serve as a compensatory mechanism to protect the maternal-fetal dyad against pathogens when the adaptive immune response is suppressed. Overall our data provide a blueprint of pregnancy induced changes in the peripheral blood transcriptome and elucidate new pathways and molecules that function in the modulation of the immune system during the third trimester of human pregnancy.

#### Oxidative stress and DNA damage

Leukocyte DNA damage was approximately two times greater among third trimester pregnant women than nonpregnant control women. This elevation in DNA damage among pregnant women may arise from increases in oxidative stress particularly as pregnancy advances [9]. Healthy pregnant women were previously thought to overcome oxidative stress through upregulation of antioxidation machinery [36], [37]. However, our study suggests that DNA damage commonly occurs throughout the last third of uncomplicated pregnancies.

It is noteworthy that oxidative stress and immune activation are interrelated. Neutrophils are both activated by, and a producer of, reactive oxygen species. TLR4 signaling is enhanced by reactive oxygen species, leading to sustained production of proinflammatory proteins, which in turn maintain oxidative stress [38]. In addition, activated neutrophils are a significant source of reactive oxygen species via the activity of NADPH oxidase and myeloperoxidase (MPO) [39], which were both upregulated among pregnant women in our study. Although the increased oxidative stress appears to be a normal event of pregnancy, the DNA damage by reactive oxygen species can lead to genomic instability, aberrant gene expression and apoptosis [40], [41]. Additional studies are needed to address the health effects of pregnancy-induced leukocyte DNA damage and whether the DNA damage resolves after delivery.

### The effects of choline intake

A higher maternal choline intake during pregnancy increased dimethylation of H3K4, which is indicative of active transcription [42]. To the best of our knowledge, this is the first report in humans that choline intake affected an epigenetic mark of histones obtained from leukocytes of pregnant women. Nevertheless, we did not observe a corresponding change in leukocyte gene expression by choline intake within the time frame of this study and no other genomic marks were affected by maternal choline intake. Thus, unlike the genomic marks (e.g., DNA methylation [15] and transcriptome profiles (unpublished data) of developing fetal-derived tissues (i.e., placenta and cord blood leukocytes) which are highly responsive to maternal choline intake, genomic markers of maternal-derived tissue may be relatively unresponsive to changes in maternal choline intake.

### Limitations

Our study has two major limitations. First, data collection was limited to the third trimester of pregnancy; thus, additional studies are needed to address the effects of pregnancy and maternal choline intake on maternal genomic marks in earlier stages of gestation as well as in the postnatal period. Second, genomic marks from peripheral blood sampling do not necessarily reflect genomic marks in other tissues. Nonetheless, blood transcript profiling is considered a robust tool for assessing the status of the human immune system [43], [44], [45].

### Conclusions

In summary, the last third of human pregnancy is characterized by transcriptional activation of the neutrophils and elevations in maternal leukocyte oxidative DNA damage. These findings are consistent with an upregulation of the peripheral innate immune system which plays a central role in protecting the maternal and fetal dyad against pathogens. Additional studies are needed to delineate the long-term functional consequences of these genomic alterations and to investigate the relationship between these genomic marks and complicated pregnancy.

## Supporting Information

Table S1 **Genes differentially expressed in third trimester pregnant women and non-pregnant women at week 0 (baseline) and week 12 (study end) of the controlled feeding study.** Benjamini-Hochberg adjusted *P* values<0.05; Data were analyzed with the LEMMA statistical package.
(XLSX)

Table S2 **Gene ontology altered in third trimester pregnant women and non-pregnant women at week 12 (study end) of the controlled feeding study.** Data were analyzed with Gominer.
(XLSX)
